# The Frontiers of Smart Healthcare Systems

**DOI:** 10.3390/healthcare12232330

**Published:** 2024-11-21

**Authors:** Nan Lin, Rudy Paul, Santiago Guerra, Yan Liu, James Doulgeris, Min Shi, Maohua Lin, Erik D. Engeberg, Javad Hashemi, Frank D. Vrionis

**Affiliations:** 1 Department of Gastroenterology, The Affiliated Hospital of Putian University, Putian 351100, China; linnan0898@163.com (N.L.); piaopiaoliu043@gmail.com (Y.L.); 2 Department of Ocean & Mechanical Engineering, Florida Atlantic University, Boca Raton, FL 33431, USA; paulr2017@fau.edu (R.P.); guerras2023@fau.edu (S.G.); eengeberg@fau.edu (E.D.E.); 3 Department of Neurosurgery, Marcus Neuroscience Institute, Boca Raton Regional Hospital, Boca Raton, FL 33486, USA; 4 Department of Biomedical Engineering, Florida Atlantic University, Boca Raton, FL 33431, USA; jdoulgerisphd@gmail.com (J.D.); jhashemi@fau.edu (J.H.); 5 Harvard Ophthalmology AI Lab, Schepens Eye Research Institute of Massachusetts Eye and Ear, Harvard Medical School, Boston, MA 02115, USA; min.shi@louisiana.edu; 6 School of Computing and Informatics, University of Louisiana, Lafayette, LA 70504, USA; 7 Center for Complex Systems and Brain Science, Florida Atlantic University, Boca Raton, FL 33431, USA

**Keywords:** Artificial Intelligence (AI), healthcare, surgery, medical imaging, smart healthcare systems

## Abstract

Artificial Intelligence (AI) is poised to revolutionize numerous aspects of human life, with healthcare among the most critical fields set to benefit from this transformation. Medicine remains one of the most challenging, expensive, and impactful sectors, with challenges such as information retrieval, data organization, diagnostic accuracy, and cost reduction. AI is uniquely suited to address these challenges, ultimately improving the quality of life and reducing healthcare costs for patients worldwide. Despite its potential, the adoption of AI in healthcare has been slower compared to other industries, highlighting the need to understand the specific obstacles hindering its progress. This review identifies the current shortcomings of AI in healthcare and explores its possibilities, realities, and frontiers to provide a roadmap for future advancements.

## 1. Introduction

The integration of Artificial Intelligence (AI) into healthcare represents one of the most transformative trends in modern medicine [1]. From administrative tasks to highly complex diagnostics and surgical interventions, AI is making significant strides in reshaping how care is delivered [2]. This transformation is driven by the ability of AI to analyze vast amounts of medical data, generate predictive insights, and facilitate decision-making processes that can enhance patient outcomes [3]. With global healthcare systems facing challenges such as rising costs, staff shortages, and the need for more personalized care, AI offers promising solutions across the board [4].

Healthcare systems around the world are burdened by inefficiencies in both administrative and clinical processes [5]. Administrative tasks, such as scheduling, billing, and record management, consume a significant portion of healthcare resources and often lead to delays or errors in patient care [6,7]. AI’s ability to automate routine tasks and manage large datasets in real time has the potential to alleviate these burdens, allowing healthcare providers to focus more on patient care rather than paperwork [8,9].

In the clinical realm, AI’s role extends far beyond mere automation. Advanced AI algorithms, particularly those based on machine learning (ML) and deep learning (DL), are being employed to improve diagnostic accuracy, streamline medical imaging, and even assist in surgical procedures [10,11]. For example, AI-driven systems have been developed to analyze medical images, detect abnormalities, and provide diagnostic recommendations that can significantly reduce the time needed to diagnose conditions such as cancer or cardiovascular diseases [12,13].

Despite its potential, AI adoption in healthcare has not progressed as rapidly as in other industries. This is due to a combination of technical, ethical, and regulatory challenges [14]. Data privacy concerns, the black-box nature of many AI models, and the need for standardized data formats all pose significant obstacles to AI’s widespread integration into healthcare systems [15]. Additionally, the inherent complexity of healthcare data, which include a mixture of structured and unstructured data, makes it difficult to develop AI models that can be generalized across different healthcare settings [16,17].

Moreover, AI applications in healthcare raise important ethical and legal questions. Who is responsible when an AI system makes a wrong diagnosis or recommendation [18]? How can we ensure that AI algorithms do not perpetuate biases present in the data they are trained on [3]? These concerns highlight the importance of developing transparent, explainable AI systems that healthcare professionals can trust [18,19].

Numerous reviews have highlighted the profound impact of artificial intelligence (AI) on healthcare, often focusing on specific areas such as medical imaging, precision medicine, or ethical considerations. For instance, researchers emphasize the convergence of AI and precision medicine, where AI’s capabilities in data processing and pattern recognition help optimize individualized treatments [20]. Similarly, they advocate for the application of AI in drug discovery, clinical trials, and patient care, identifying AI’s role in automating tasks and improving data-driven decisions [21].

Recent review papers provide valuable insights into AI applications, challenges, and future directions. One paper surveys current AI applications and discusses techniques such as machine learning and natural language processing, particularly in major disease areas like cancer, neurology, and cardiology [22]. Another review offers a comprehensive analysis of AI and machine learning applications across various healthcare sectors, including diagnostics, predictive analytics, personalized medicine, and administrative tasks [23].

Another paper discusses the challenges facing global health systems, such as aging populations and the rise of chronic diseases, positioning AI as a promising solution [24]. Lastly, another review tackles critical issues like ethical considerations, algorithmic biases, interpretability, regulatory constraints, and integration challenges that hinder AI adoption. It underscores the necessity for collaboration among healthcare practitioners, technologists, regulators, and ethicists to overcome these obstacles [25].

Other reviews focus on AI’s economic impact in healthcare systems, underscoring its potential for cost-effectiveness, but also noting the current limitations in economic assessment methodologies [26]. Ethical challenges, including bias, accountability, and privacy concerns, have also been extensively reviewed, with calls for governance frameworks to mitigate risks [27].

What distinguishes this review from previous work is its comprehensive approach to assessing AI’s role in multiple healthcare domains—from administration and diagnostics to interventions—while simultaneously addressing the unique technical, ethical, and implementation challenges that impede large-scale AI adoption. This review goes beyond focusing on individual AI applications to provide a holistic perspective on the integration of AI into healthcare systems, identifying common shortcomings and proposing future directions that align AI’s development with healthcare’s evolving needs.

**Figure 1:**
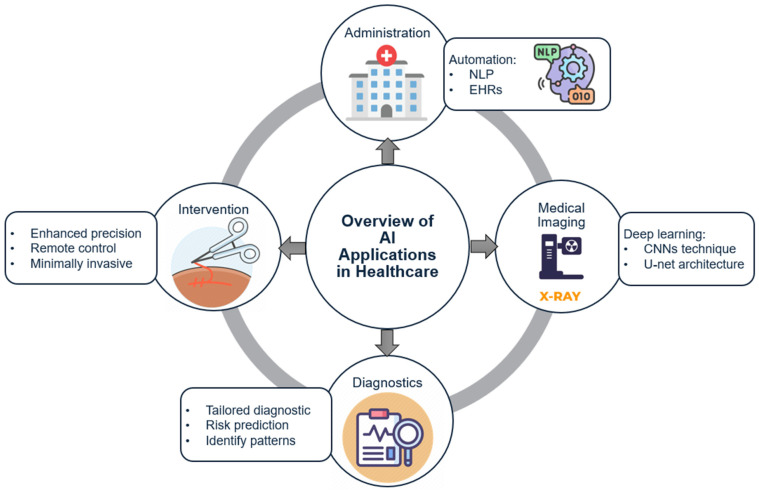
Overview of AI Applications in Healthcare.

In this review, we explore the current applications of AI in healthcare, focusing on key areas such as administrative processes, medical imaging, diagnostics, and surgical interventions. We also examine the challenges that must be overcome to fully realize the potential of AI in this domain and discuss the future frontiers of smart healthcare systems. By addressing both the possibilities and limitations of AI, this paper aims to provide a comprehensive understanding of how AI can revolutionize healthcare in the coming years (Figure 1).

## 2. Methods

This review was conducted to provide a comprehensive and structured analysis of AI applications in healthcare. The research process involved a multi-step method of literature selection, data extraction, and thematic analysis to ensure thorough coverage of the different dimensions of AI in healthcare [28]. Below are the key steps in the methodological process used in this review.

### 2.1. Literature Search Strategy

A systematic literature search was conducted across major scientific databases, including **PubMed**, **IEEE Xplore**, **Google Scholar**, **Scopus**, and **Web of Science**. The primary aim was to identify peer-reviewed studies, reviews, case studies, and industry reports related to AI applications in healthcare. The search included publications from **2015 to 2024**, capturing recent advancements as well as foundational studies.

Key search terms included a combination of the following:

- “Artificial Intelligence in Healthcare”;
- “AI in Medical Imaging”;
- “AI Diagnostics”;
- “Robotic Surgery”;
- “Smart Healthcare Systems”;
- “AI and Electronic Health Records”;
- “Predictive Analytics in Healthcare”.

Boolean operators, such as “AND”, “OR”, and “NOT”, were used to narrow or broaden the search criteria as appropriate. For example, searches such as “AI AND Medical Imaging” AND “U-Net” or “Deep Learning AND Healthcare” were performed to capture focused results.

### 2.2. Selection Criteria

To ensure that only relevant, high-quality studies were included, the following selection criteria were applied:

**A.**  **Inclusion Criteria:**
  ○ Studies that directly addressed the use of AI in one of the following healthcare domains: administration, medical imaging, diagnostics, or surgical interventions.
  ○ Articles published between **2015 and 2024** to cover both foundational AI technologies and cutting-edge advancements.
  ○ Peer-reviewed journal articles, case studies, and industry white papers that provide empirical evidence or theoretical discussions on AI applications.
  ○ Studies with at least an abstract and full text available in English.
**B.**  **Exclusion Criteria:**
  ○ Studies focusing solely on AI theory without direct healthcare application.
  ○ Articles published before 2015 unless they provided foundational AI methodologies that are still relevant.
  ○ Non-peer-reviewed sources that lacked empirical data, including news articles, editorials, or opinion pieces.

The selection process involved screening the titles and abstracts of articles first. Full-text review was conducted for those studies that passed the initial screening phase. Any articles that did not meet the inclusion criteria were excluded.

### 2.3. Data Extraction

The data extraction process was designed to ensure consistency and comprehensiveness. For each selected study, the following information was extracted:

- **Author**(**s**) **and Year of Publication**: to track the development of AI technologies over time.
- **Study Type**: whether it was an experimental study, a review article, a case study, or an industry report.
- **Healthcare Domain**: categorized into administration, imaging, diagnostics, or intervention.
- **AI Technology Used**: such as machine learning algorithms (CNNs, RNNs), deep learning models (U-Net, GANs), or NLP tools.
- **Outcome and Findings**: summarized to highlight the main contributions of the study, including any limitations or challenges noted by the authors.
- **Challenges Identified**: particularly focusing on barriers to AI implementation such as ethical concerns, data privacy, and algorithmic bias.

### 2.4. Thematic Analysis

A thematic analysis was conducted to identify common trends, challenges, and outcomes across the studies. The studies were grouped by healthcare domain to assess the specific role of AI in that area. Within each domain, key themes such as “AI model accuracy”, “operational efficiency”, “ethical challenges”, and “cost-effectiveness” were identified and compared.

The thematic analysis allowed for a synthesis of the findings across multiple studies, providing a comprehensive overview of the state of AI in healthcare and highlighting areas for future research and development.

## 3. Results

### 3.1. Limitations of Healthcare

Healthcare systems across the globe face a multitude of challenges, including administrative inefficiencies, diagnostic errors, high costs, and a shortage of skilled professionals [29]. AI holds promise for alleviating many of these challenges, but several shortcomings must be addressed before its potential can be fully realized [30].

**A.**  **Administration**

Administrative inefficiencies have long plagued healthcare systems. From appointment scheduling to billing and patient record management, administrative tasks are often cumbersome and error-prone [31]. Paperwork can lead to mistakes and diminish operational effectiveness. The integration of AI with legacy healthcare systems is a major challenge. Many healthcare institutions still use outdated software and hardware, making it difficult to implement AI solutions. Data privacy and security are also major concerns, as healthcare data are highly sensitive and subject to strict regulations such as the Health Insurance Portability and Accountability Act (HIPAA) [32].

Furthermore, healthcare institutions must address patients’ common questions, provide education, and offer support, all of which enhance patient satisfaction and alleviate the workload for medical staff [33]. With healthcare data growing at an exponential rate, there is a pressing need to organize and manage information efficiently [34]. The World Health Organization (WHO) has highlighted that inadequate data management can lead to critical errors, delays in patient care, and increased healthcare costs [35].

Interoperability is another significant barrier to the effective use of AI in healthcare administration. The lack of standardized data formats and protocols makes it difficult for AI systems to communicate with existing EHRs, resulting in data fragmentation and inefficiencies [36].

**B.**  **Imaging**

**Figure 2:**
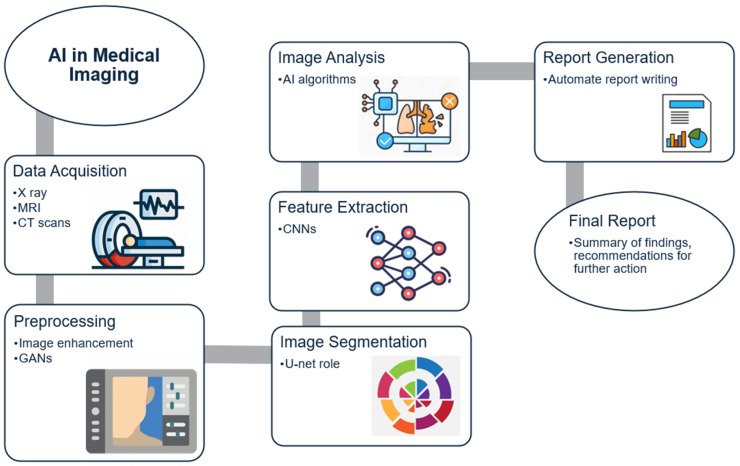
AI in Medical Imaging Workflow.

Medical imaging is one of the most important diagnostic tools in modern medicine (Figure 2). Technologies like X-ray, MRI, and CT scans are critical for detecting and diagnosing a wide range of conditions [37]. Table 1 shows a summary of AI technologies in healthcare applications. However, medical imaging is expensive and requires highly specialized skills, making it inaccessible to many patients in low-resource settings [38]. AI presents an opportunity to reduce the cost and increase the accessibility of medical imaging [39].

AI in medical imaging faces challenges related to data variability and generalizability. Imaging data from different institutions may vary significantly due to differences in equipment, imaging protocols, and patient populations, making it difficult for AI models to perform consistently across different settings [40]. Additionally, the “black box” nature of many AI models makes it difficult to understand how they arrive at their conclusions, which hinders trust and adoption [7].

**Table 1:** Summary of AI Technologies in Healthcare Applications.

AI Technology	Description	Application in Healthcare	Advantages	Challenges
CNN [41]	Deep learning architecture for analyzing visual data	Medical Imaging	High accuracy in image analysis	Requires large training datasets
NLP [42]	Analyzing and processing human language	Administration, EHR	Automates data transcription	Issues with language variability
GAN [43]	Neural networks for generating new data	Image Augmentation	Enhances model training	Computationally expensive
RNN [44]	Neural networks for processing sequential data	Patient Monitoring, Predictive Analytics	Effective for time-series analysis	Difficulty in training on long sequences
Reinforcement Learning [45]	Learning through trial and error to maximize outcomes	Treatment Planning	Personalized treatment recommendations	Complex to implement in clinical settings
Decision Trees [46]	Simple model that splits data into branches for decision-making	Risk Assessment	Easy to interpret and visualize	Prone to overfitting
Support Vector Machines [47]	Supervised learning model for classification tasks	Diagnostics	Effective in high-dimensional spaces	Requires careful tuning of parameters

Challenges remain in achieving consensus on methodologies and algorithms used in medical imaging [18,40]. In addition, medical imaging data often contains noise and various forms of attenuation, as well as motion devices. Techniques such as MRI, CT, ultrasound, and positron emission tomography (PET) are particularly susceptible to multiplicative noise [48].

**C.**  **Diagnostics**

Diagnostics is an area where AI can make a significant impact, particularly in reducing diagnostic errors and enabling early detection of diseases. However, applying AI in diagnostics is complex and fraught with challenges [49]. The use of AI in diagnostics requires large, high-quality datasets, but data variability across hospitals poses a significant barrier [50]. For example, differences in preparation and staining procedures for tissue samples create variation that affects the accuracy of AI models [51].

Furthermore, dataset bias is a critical concern in AI diagnostics. If the training data are not representative of diverse populations, the model’s performance may vary significantly across different demographic groups. This could lead to disparities in healthcare outcomes and exacerbate existing inequalities [52,53].

For example, a study evaluated how biased AI models affect clinicians’ diagnostic accuracy. Conducted from April 2022 to January 2023 across 13 U.S. states, the study involved hospital-based physicians, nurse practitioners, and physician assistants who reviewed nine clinical cases of acute respiratory failure. Clinicians first reviewed two cases without AI assistance to establish baseline accuracy (73.0%), then assessed six cases with AI predictions, either standard or biased, and with or without explanations. Standard AI models improved accuracy by 2.9 percentage points (4.4 with explanations). In contrast, biased AI predictions decreased accuracy by 11.3 points, and explanations only slightly mitigated this, improving accuracy by a non-significant 2.3 points. Thus, standard AI enhanced accuracy, but biases in AI reduced it significantly [54].

**Figure 3:**
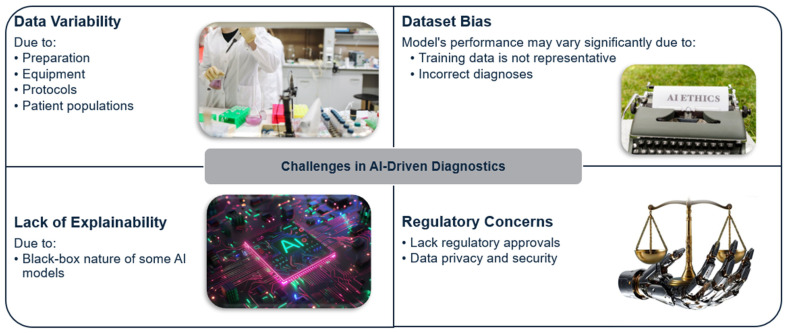
Challenges in AI-Driven Diagnostics.

The main challenge in using AI for diagnostics is ensuring transparency and explainability (Figure 3). Many AI models are complex and difficult to interpret, making it challenging for healthcare professionals to trust their recommendations. Ethical issues also arise when relying too heavily on AI for decision-making, particularly in cases where the model’s recommendations may be incorrect or biased [51]. These issues need to be addressed through rigorous validation, transparency, and clear regulations [55,56].

**D.**  **Intervention**

The adoption of AI-driven surgical systems is limited by high costs, regulatory challenges, and the need for extensive training for surgeons. The cost of acquiring and maintaining robotic systems is prohibitive for many healthcare institutions, particularly in low- and middle-income countries [57]. Table 2 shows current and future AI applications in healthcare. In addition, integrating AI into the procedural workflow requires extensive training for surgeons, and there are concerns regarding the reliability and safety of autonomous surgical procedures, as AI-driven systems may not be able to handle unexpected situations as effectively as human surgeons [56].

The regulatory approval process for AI-driven surgical systems is stringent, as patient safety is of paramount importance. The FDA and other regulatory bodies require extensive testing and validation before approving AI systems for use in surgery, which can be time-consuming and costly [56,58].

### 3.2. Possibilities of AI in Healthcare

AI holds immense potential to address many of the challenges faced by healthcare systems today. This section explores the various possibilities for AI across different aspects of healthcare.

**A.**  **Administration**

AI has the potential to revolutionize healthcare administration by automating repetitive tasks and enabling predictive analytics. For instance, NLP can be used to extract meaningful information from unstructured clinical notes and convert it into structured data for use in EHRs [22]. This would not only reduce the burden on healthcare professionals but also improve data quality and accessibility [59,60,61]. However, integrating AI into existing healthcare systems remains a major challenge. The lack of standardized data formats, data privacy concerns, and interoperability issues are significant obstacles [62]. According to a study, over 60% of healthcare institutions report difficulties in integrating AI into their existing infrastructure due to these issues [32].

Predictive analytics is another area where AI can make a difference. By analyzing patient data, AI can predict hospital readmissions, optimize resource allocation, and identify patients at risk of developing chronic conditions [63]. This can help healthcare providers intervene early and prevent adverse outcomes, ultimately reducing healthcare costs [64].

**B.**  **Imaging**

AI has the potential to significantly improve medical imaging by reducing the time and cost associated with image interpretation. AI algorithms can assist radiologists by identifying abnormalities, providing second opinions, and even generating synthetic training data to improve model accuracy [65]. Techniques like GANs have been used to enhance image quality and create realistic images for training purposes, which is particularly useful when dealing with rare conditions [66].

AI can also assist in early disease detection. For instance, AI has been used to detect diabetic retinopathy from retinal images with high accuracy, allowing for early intervention and preventing vision loss [67]. By augmenting the capabilities of radiologists, AI can improve diagnostic accuracy and reduce the workload on healthcare professionals [68].

Deep learning techniques, particularly convolutional neural networks (CNNs), have shown promise in interpreting medical images and assisting radiologists [69]. U-Net is one of the architectures that has gained traction for image segmentation tasks, providing accurate delineations of tumors and other abnormalities [70]. Table 3 illustrates the challenges and solutions for AI adoption in healthcare.

**C.**  **Diagnostics**

The use of AI in diagnostics has the potential to revolutionize personalized medicine. By analyzing a patient’s genetic information, medical history, and clinical data, AI can provide tailored diagnostic recommendations and treatment plans [73]. For example, AI has been used to predict the risk of developing certain cancers based on genetic markers and lifestyle factors [74].

Moreover, AI models can identify patterns in medical data that may not be apparent to human practitioners. Machine learning algorithms have been used to predict sepsis, a life-threatening condition, hours before it becomes clinically apparent, allowing for timely intervention [75,76]. These advancements in AI-driven diagnostics have the potential to save lives and improve patient outcomes [76].

**D.**  **Intervention**

AI-driven surgical systems are being developed to enhance the precision and efficacy of surgical procedures. One example is the use of AI in real-time decision support during surgery [77]. AI can analyze data from various sources, such as imaging and sensors, to provide surgeons with actionable insights during procedures. This can help improve surgical outcomes and reduce the risk of complications [78].

The concept of remote surgery, or teleoperation, is also being explored with the help of AI. By using AI to enhance remote control of surgical robots, surgeons can perform procedures on patients located in remote or underserved areas, expanding access to specialized care [79]. This has the potential to address healthcare disparities and provide high-quality care to patients who would otherwise lack access [79].

### 3.3. Realities of AI in Healthcare

While AI holds immense potential, its adoption in healthcare is still in its early stages. This section discusses the current realities and limitations of AI in different aspects of healthcare.

**A.**  **Administration**

AI has already made significant inroads into healthcare administration, automating tasks such as appointment scheduling, medical coding, and billing [64]. Chatbots powered by AI are also being used to engage with patients, answer queries, and provide information [80]. However, challenges remain in ensuring data privacy and security, as healthcare data are highly sensitive and subject to strict regulations [32].

A nowadays example of the use of AI in administration is Nuance’s Computer-Assisted Physician Documentation™ (CAPD) system at Universal Health Services (UHS), which led to significant improvements, including a 69% reduction in transcription costs (saving $3 million annually), a 12% increase in the case mix index (CMI) for resource allocation, a 36% improvement in documenting severe illness cases, and a 24% increase in detail for high-risk patients. The cloud-based speech CAPD system and the Clinical Documentation Improvement (CDI) workflow at UHS supports physician engagement in quality improvement by addressing challenges in documentation, reducing transcription costs, speeding up documentation, and enhancing the accuracy and detail of patient records to improve quality metrics and reimbursements [81].

AI systems need to be integrated with existing EHRs, but the lack of standardized data formats and protocols makes this difficult [82]. A study found that data fragmentation and a lack of interoperability are significant barriers to the effective use of AI in healthcare administration [36].

**B.**  **Imaging**

AI is currently being used in medical imaging to assist radiologists in detecting abnormalities and providing second opinions [83]. For instance, AI algorithms have been used to detect lung nodules in CT scans, with some studies reporting performance comparable to human radiologists [84].

For example, a study evaluated whether an AI-based computer-aided detection (AI-CAD) software could reduce false positives per image (FPPI) in mammograms compared to a conventional FDA-approved CAD. Conducted retrospectively on 250 mammograms from early 2013, it compared the two systems’ sensitivity and specificity in cancer detection, focusing on the number of false-positive marks per image and completely mark-free cases. Results indicated a significant 69% overall reduction in FPPI with AI-CAD, with reductions of 83% for calcifications and 56% for masses, while maintaining sensitivity. Nearly half (48%) of cases had no marks with AI-CAD, compared to only 17% with conventional CAD. This reduction in false positives could also reduce radiologist reading time by 17% per case, providing social and economic benefits by decreasing unnecessary recalls in screening [85].

Recent studies highlight AI’s potential in enhancing post-operative evaluations and implant selection in spinal surgeries. For instance, Lin et al. developed a 3D-printed cervical spine model embedded with a sensor array to evaluate intervertebral pressure distributions, offering precise, patient-specific insights that aid in minimizing complications like implant misalignment and degeneration [86]. Another study by Lin et al. explored a robotic spine replica with a soft magnetic sensor array to simulate various postures and forecast post-operative outcomes, leveraging machine learning to enhance accuracy in force and posture prediction [87]. A recent study on a soft robotic hand exoskeleton for music training showcases how AI-driven feedback systems can aid in the rehabilitation of patients with motor impairments (Lin et al., 2023). This exoskeleton, equipped with tactile sensors and machine learning algorithms, enabled patients to relearn fine motor skills, such as playing musical instruments, by distinguishing correct from incorrect actions with high accuracy. The exoskeleton’s AI capabilities provide real-time feedback to patients, significantly enhancing motor recovery and skill reacquisition, especially for those recovering from neurotrauma [88]. These examples underscore how AI-driven models and sensor technologies can support personalized treatment plans, ultimately improving surgical precision and patient outcomes by providing data-driven guidance for clinicians.

Despite these advancements, the adoption of AI in medical imaging is limited by the need for human oversight and regulatory approval [89]. Another limitation is the variability in imaging data. AI models trained on data from one institution may not perform well when applied to data from another institution due to differences in equipment, imaging protocols, and patient populations [40]. This lack of generalizability hinders the widespread adoption of AI in medical imaging [90]. Table 4 lists the AI models used in medical imaging.

**C.**  **Diagnostics**

AI-driven diagnostic tools are being used for disease detection and predictive analytics, but their adoption is still limited. Most AI diagnostic systems require validation through clinical trials, which can be time-consuming and expensive [91]. Additionally, ethical concerns related to accountability and bias need to be addressed before AI can be fully trusted for diagnostic decision-making [51]. If the training data are not representative of the entire population, the model may perform poorly for certain demographic groups, leading to disparities in healthcare outcomes [52,53]. Table 5 provides some ethical and regulatory considerations for AI in healthcare.

**Table 4:** AI Models Used in Medical Imaging.

AI Model	Application	Advantages	Limitations
U-Net [92]	Tumor Segmentation	High accuracy in delineation	Requires large datasets
GAN	Image Augmentation	Effective in improving model performance	Computationally intensive
VGGNet [93]	Image Classification	Strong feature extraction	Deep architecture, Overfitting risk
ResNet [94]	Image Classification	Addresses vanishing gradient problem	Complexity increases with depth
DenseNet [95]	Lesion Detection	Efficient feature reuse	High memory consumption
YOLO [96]	Object Detection	Real-time processing capability	Less accuracy for small objects
Xception [97]	Disease Classification	Depthwise separable convolutions for efficiency	Requires extensive tuning
MobileNet [98]	Mobile Imaging Applications	Lightweight and fast for mobile devices	Lower accuracy compared to larger models
Faster R-CNN [99]	Tumor Detection	High accuracy in detection	Slower than single-shot models

**D.**  **Intervention**

AI-driven surgical systems, such as the da Vinci system, have demonstrated their ability to enhance the precision of minimally invasive surgeries. Another notable example is the MAKO robotic system, a premier platform for orthopedic surgeries; the system enhances alignment and improves accuracy in component placement, leading to better implant survival rates and a reduction in revision surgeries [100]. These technologies allow surgeons to perform complex tasks with greater accuracy, reducing the risk of complications and improving patient outcomes [101].

**Figure 4:**
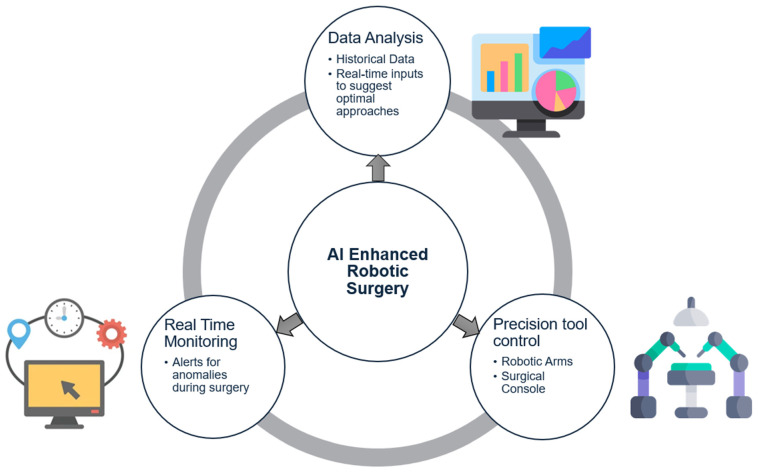
AI-Enhanced Robotic Surgery.

However, the adoption of these systems is limited by high costs, regulatory challenges, and the need for extensive training for surgeons [101]. There are also concerns about the reliability and safety of autonomous surgical procedures, as AI-driven systems (Figure 4) may not be able to handle unexpected situations as effectively as human surgeons [7].

The regulatory approval process for AI-driven surgical systems is stringent, as patient safety is of paramount importance. The FDA and other regulatory bodies require extensive testing and validation before approving AI systems for use in surgery [56]. This process can be time-consuming and costly, delaying the adoption of AI in surgical intervention.

### 3.4. Frontiers of Smart Healthcare

**Figure 5:**
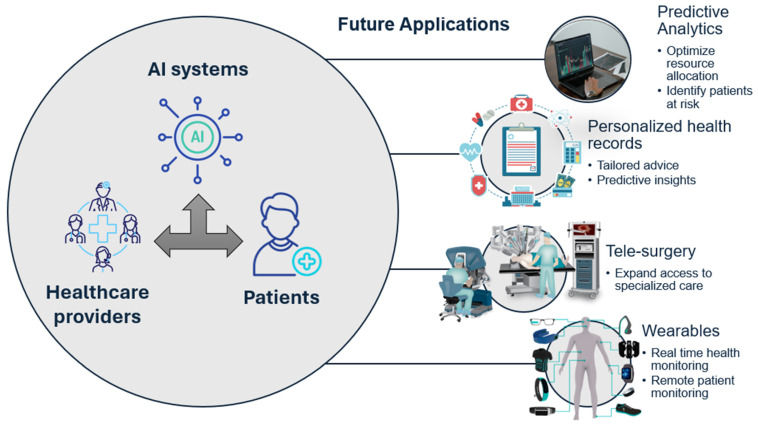
Future Applications of AI in Smart Healthcare.

The future of smart healthcare lies in advancing AI to address current challenges and realize its transformative potential. This section explores the frontiers of AI in different aspects of healthcare (Figure 5).

**A.**  **Administration**

The future of healthcare administration could involve AI-driven personalized health records, where AI not only stores patient data but actively provides tailored healthcare advice and predictive insights for individual patients. AI could also be used to predict healthcare trends, such as disease outbreaks, and help healthcare providers allocate resources accordingly [32].

Blockchain technology could be integrated with AI to ensure the security and privacy of patient data. By using blockchain to create a decentralized and secure system for storing patient information, healthcare institutions could address many of the data privacy concerns associated with AI [102].

**B.**  **Imaging**

Next-generation AI-augmented imaging systems could allow for real-time imaging during surgeries, enabling surgeons to make data-driven decisions on the spot. AI could also be used to create personalized imaging protocols based on a patient’s medical history, improving the accuracy of diagnoses [40].

AI may also enable faster and more accessible imaging for underserved populations by reducing the cost of imaging equipment. Portable AI-powered imaging devices could be used in remote areas to provide high-quality diagnostic imaging, improving access to healthcare for patients who would otherwise lack it [65].

**C.**  **Diagnostics**

AI has allowed more effective Internet of Things (IoT) solutions that hold the potential to deliver self-diagnostic tools to patients, allowing them to monitor their health via smartphones and wearables. By combining real-time data with advanced AI models, personalized, preventative care could become a reality [8].

AI could also be used to develop predictive models that identify patients at risk of developing chronic conditions, such as diabetes or cardiovascular disease. By analyzing genetic information, lifestyle factors, and clinical data, AI could provide personalized recommendations for preventing the onset of these conditions [75].

**D.**  **Intervention**

Surgical intervention is another area where AI has shown potential, particularly with the rise of robotic-assisted surgery. Robotic systems, such as the da Vinci Surgical System, can provide enhanced precision, dexterity, and control during minimally invasive procedures [103]. The MAKO robotic system is a leading platform for orthopedic surgeries that can also benefit from AI, since it utilizes preoperative CT scans for detailed surgical planning and provides haptic feedback during procedures to ensure precise bone resection [104].

The frontier of AI in surgical intervention includes fully autonomous surgical robots capable of handling high-risk procedures with minimal human intervention. AI’s role in enhancing tele-surgery capabilities could make specialized care available globally, especially in remote regions [105].

AI-driven rehabilitation is another promising area of development. By using AI to analyze a patient’s progress and adjust therapy in real-time, personalized rehabilitation programs could be created to optimize recovery and improve patient outcomes [106].

## 4. Discussion

### 4.1. Overcoming Shortcomings in Healthcare with AI

AI has the potential to resolve many of healthcare’s inefficiencies, especially in administration, imaging, diagnostics, and surgeries. However, barriers such as data standardization, privacy concerns, and high costs must be addressed before AI can reach its full potential [107]. Integrating AI into administrative tasks requires improvements in infrastructure, interoperability, and secure data management [108].

In imaging and diagnostics, AI models struggle with data variability and bias, which limits their ability to generalize across different patient populations [109]. Additionally, the high costs and training demands of AI-driven surgical systems pose challenges, particularly for hospitals in low-resource settings. Overcoming these obstacles will require more affordable technologies and stronger regulatory frameworks [110].

### 4.2. Realizing the Potential of AI

AI’s transformative potential in healthcare lies in its ability to automate tasks, improve diagnostic accuracy, and optimize resource management. In administration, AI can reduce human errors and free up healthcare professionals to focus more on patient care. Predictive analytics could help hospitals better manage patient flow and resources, while in medical imaging, AI can assist radiologists in detecting abnormalities more quickly and accurately. AI also holds promise for personalized medicine, providing tailored treatment recommendations based on individual data. Furthermore, advances in tele-surgery and AI-assisted rehabilitation could expand access to specialized care in underserved regions. To unlock this potential, collaboration between AI developers, healthcare providers, and regulators is essential to ensure successful and ethical implementation.

### 4.3. Ethical and Legal Considerations

As AI’s role in healthcare grows, so too will the ethical and legal questions surrounding its use. One of the most pressing concerns is the issue of accountability. Who is responsible when an AI system makes an incorrect diagnosis or surgical decision? As AI systems become more autonomous, legal frameworks must be established to clarify liability in cases of malpractice or error [19,111]. Moreover, transparency is essential. AI developers must focus on creating explainable systems that allow healthcare professionals to understand and validate the decisions made by AI models.

Bias in AI models is another crucial issue, as biased algorithms can lead to disparities in healthcare outcomes. Addressing this problem requires the development of unbiased, transparent AI systems, along with policies that ensure fairness and equity in AI-driven healthcare services [112]. Interdisciplinary collaboration between AI developers, healthcare providers, regulators, and ethicists will be necessary to ensure that AI applications in healthcare are not only technologically sound but also ethically responsible.

### 4.4. The Road Ahead: Future Directions

The future of AI in healthcare is promising, but several challenges must be overcome to unlock its full potential. First, the issue of generalizability and scalability of AI models must be addressed. This will require large, diverse datasets that represent the global patient population. Additionally, there is a need for increased collaboration between healthcare providers, AI researchers, and regulatory bodies to create standardized, validated AI systems that can be implemented safely and effectively across various healthcare settings [113].

The development of explainable AI (XAI) systems is also a critical next step. AI models must be interpretable and transparent to build trust among healthcare professionals and patients alike. Furthermore, AI developers must focus on creating systems that not only assist but also complement human decision-making, allowing healthcare providers to make informed, data-driven decisions with confidence.

By addressing these challenges and fostering collaboration across industries and sectors, AI has the potential to revolutionize healthcare, leading to improved patient outcomes, greater accessibility, and more efficient care.

## 5. Conclusions

The integration of AI in healthcare requires a clear regulatory framework and standardized practices to ensure its safe and effective application. Policymakers should establish specific guidelines for data privacy, algorithm certification, and explainable AI to support transparency and accountability. Standardizing certification for AI algorithms, similar to medical device protocols, will ensure these tools meet accuracy and reliability benchmarks before clinical use. Additionally, healthcare organizations should incorporate ongoing monitoring, regular audits, and updates to maintain AI effectiveness. Promoting cross-institutional data sharing while protecting privacy will strengthen AI models, and collaboration across stakeholders—including developers, clinicians, and ethicists—will address ethical concerns. Finally, AI education and training should be embedded in medical curricula to prepare healthcare professionals for AI-enabled care. These measures can facilitate responsible AI implementation that enhances patient outcomes and trust in healthcare innovation.

## Figures and Tables

**Table 2:** Current and Future AI Applications in Healthcare.

Healthcare Domain	Current Applications	Future Applications
Administration	Chatbots for patient inquiries Scheduling automation	Personalized health records Predictive resource management
Imaging	AI-assisted image analysis Tumor segmentation	Real-time imaging during surgery Portable AI-powered imaging devices
Diagnostics	AI-driven disease detection, Risk prediction	Self-diagnosis tools Preventative care systems
Surgical Intervention	Robotic-assisted surgery (e.g., da Vinci, MAKO)	Fully autonomous surgical robots AI-enhanced tele-surgery
Patient Monitoring	Wearable devices for health tracking	Continuous remote monitoring with AI alerts
Drug Development	AI for drug discovery and development	AI-driven personalized medicine Treatment optimization
Mental Health	AI chatbots for mental health support	Predictive analytics for mental health crises
Telemedicine	Virtual consultations with AI-assisted triage	Fully AI-integrated telehealth platforms
Public Health	Data analysis for outbreak tracking	Predictive modeling for epidemic prevention

**Table 3:** Challenges and Solutions for AI Adoption in Healthcare.

Challenge	Description	Proposed Solutions
Data Privacy	Concerns over the security of patient data	Implementing blockchain for secure data storage Strengthening data encryption
Dataset Bias	Lack of diverse training data leading to biased AI models	Ensuring diverse datasets Conducting bias audits
Lack of Explainability	Difficulty in interpreting AI model decisions	Developing interpretable AI models Using explainable AI (XAI) techniques [71]
Integration Issues	Difficulty integrating AI with legacy systems	Use of APIs for compatibility [72] Gradual system modernization
Regulatory Compliance	Navigating complex regulations for AI in healthcare	Collaborating with regulators Staying updated on guidelines
High Costs	Significant investment required for AI technologies	Leveraging cloud-based solutions Exploring public-private partnerships
Staff Resistance	Reluctance among healthcare staff to adopt new technologies	Providing training programs Highlighting AI benefits
Limited Infrastructure	Inadequate technological infrastructure to support AI	Investing in IT upgrades Utilizing cloud computing resources
Data Interoperability	Challenges in sharing and accessing patient data across systems	Adopting standardized data formats Implementing health information exchanges

**Table 5:** Ethical and Regulatory Considerations for AI in Healthcare.

Consideration	Challenges	Proposed Solutions
Accountability	Determining responsibility in case of errors	Establishing clear guidelines on liability Involving human oversight in decision-making
Patient Consent	Ensuring informed consent for AI use	Transparent communication Opt-in policies for patients
Data Security	Protection of sensitive patient information	Encryption Blockchain for data integrity
Bias and Fairness	Risk of biased AI models affecting outcomes	Diverse training datasets Regular bias audits
Transparency	Difficulty in explaining AI decision-making	Development of explainable AI (XAI) frameworks
Regulatory Compliance	Navigating complex regulatory environments	Active collaboration with regulatory bodies Staying updated on changing regulations
Privacy	Managing personal health information in AI systems	Implementing strict data access controls Anonymizing data
Trust	Building trust in AI systems among healthcare providers and patients	Educational initiatives Demonstrating efficacy through studies
Interoperability	Challenges in integrating AI with existing systems	Use of standardized data formats APIs for compatibility
